# Impact of vasopressin-assisted laparoscopic endometrioma excision on ovarian reserve: a randomized controlled trial

**DOI:** 10.3389/frph.2026.1906041

**Published:** 2026-10-02

**Authors:** Jaehoon Lee, Eunhyang Park, Hyemin Park, Inha Lee, Young Sik Choi, SiHyun Cho, Sang Wun Kim

**Affiliations:** 1 Department of Obstetrics and Gynecology, Gangnam Severance Hospital, Yonsei University College of Medicine, Seoul, Republic of Korea; 2 Institute of Women's Life Medical Science, Yonsei University College of Medicine, Seoul, Republic of Korea; 3 Department of Pathology, Severance Hospital, Yonsei University College of Medicine, Seoul, Republic of Korea; 4 Department of Obstetrics and Gynecology, Severance Hospital, Yonsei University College of Medicine, Seoul, Republic of Korea

**Keywords:** anti-Müllerian hormone, endometrioma, endometriosis, laparoscopy, ovarian reserve, ovarian surgery, vasopressin

## Abstract

**Introduction:**

Laparoscopic cystectomy is the standard surgical treatment for ovarian endometrioma; however, inadvertent removal of healthy ovarian tissue and thermal injury during hemostasis may compromise ovarian reserve. Vasopressin injection has been proposed as a surgical adjunct to reduce bleeding and limit the need for electrocoagulation, thereby potentially minimizing ovarian damage. However, evidence regarding its effectiveness in preserving ovarian reserve remains limited.

**Methods:**

This randomized controlled trial was conducted at a tertiary university-affiliated medical center between September 2018 and July 2021. Seventy-five women undergoing laparoscopic cystectomy for unilateral ovarian endometrioma were stratified according to baseline serum anti-Müllerian hormone (AMH) levels and randomly assigned to either a vasopressin-assisted surgery group or a control group. The primary outcome was the change in serum AMH level 12 months after surgery. Secondary outcomes included operative parameters, including cyst stripping time and time required for bleeding control. Outcomes were analyzed using intention-to-treat and per-protocol approaches. Longitudinal changes in AMH were evaluated using linear mixed-effects models.

**Results:**

In the intention-to-treat analysis, vasopressin injection significantly reduced the time required for cyst stripping (8.9 ± 7.9 vs. 14.4 ± 13.7 min, *p* = 0.054) and bleeding control (2.2 ± 2.5 vs. 6.5 ± 6.8 min, *p* = 0.002) compared with the control group. However, the decline in AMH levels at 12 months after surgery did not differ significantly between the vasopressin and control groups (−0.96 ± 1.11 vs. −1.14 ± 0.92 ng/mL, *p* = 0.488). Subgroup analyses according to baseline AMH levels (<3 ng/mL and 3.0–<7.0 ng/mL) yielded similar findings. Linear mixed-effects modeling demonstrated a significant decline in AMH at 3 months after surgery, followed by partial recovery at 6 and 12 months, with no significant group-by-time interaction (*p* = 0.816). Per-protocol analyses produced consistent results. Histopathological evaluation revealed no significant between-group differences in the thickness or proportion of excised ovarian tissue.

**Conclusion:**

Although vasopressin reduced operative time and electrocoagulation exposure during endometrioma surgery, it did not prevent postoperative decline in ovarian reserve.

## Plain language summary

Ovarian endometrioma surgery may reduce ovarian reserve because normal ovarian tissue can be inadvertently removed or damaged during cyst excision and hemostasis. Vasopressin is commonly used during laparoscopic surgery to reduce bleeding and the need for electrocautery. In this randomized controlled trial, women who underwent vasopressin-assisted laparoscopic excision of unilateral ovarian endometriomas experienced shorter cyst stripping and electrocoagulation times. However, ovarian reserve, assessed using serum anti-Müllerian hormone (AMH) levels over 12 months, declined similarly in both the vasopressin and control groups, regardless of baseline ovarian reserve. These findings suggest that while vasopressin may improve surgical efficiency, it does not appear to provide additional protection against postoperative ovarian reserve decline.

## Introduction

Endometriosis is a chronic inflammatory disease characterized by the presence of endometrial-like tissue outside the uterine cavity. Ovarian endometrioma is one of the most common manifestations of endometriosis, affecting approximately 17%–44% of women with the disease (1, 2). Endometriomas are of particular clinical importance in reproductive-age women because both the lesion itself and its treatment have been associated with impaired fertility and diminished ovarian reserve (3).

Although hormonal therapies are effective for symptom control and recurrence prevention, they do not reliably eliminate existing endometriomas (4). Consequently, laparoscopic cystectomy remains the standard surgical treatment for symptomatic or large endometriomas. However, surgical excision may inadvertently remove healthy ovarian tissue or cause thermal injury during hemostasis, resulting in a postoperative decline in ovarian reserve (5).

Several surgical strategies have been proposed to minimize ovarian damage during cystectomy. Among them, injection of diluted vasopressin into the cyst wall before dissection has gained attention. By creating hydrostatic separation between the cyst capsule and normal ovarian stroma, vasopressin may facilitate identification of the correct dissection plane and reduce inadvertent excision of healthy ovarian tissue (6–9). In addition, its potent vasoconstrictive effect may reduce intraoperative bleeding and the need for bipolar electrocoagulation, thereby limiting thermal damage to the ovarian cortex (6).

Despite these theoretical advantages, the effect of vasopressin-assisted cystectomy on postoperative ovarian reserve remains controversial. Previous studies have reported inconsistent findings, and substantial heterogeneity exists regarding patient characteristics, laterality of endometriomas, surgical techniques, and methods used to assess ovarian reserve (6–9, 27). Therefore, further evidence from well-defined patient populations is needed.

In the present randomized controlled trial, we evaluated whether vasopressin injection during laparoscopic cystectomy could reduce postoperative ovarian reserve loss in women with unilateral ovarian endometriomas. Serum anti-Müllerian hormone (AMH), a well-established marker of ovarian reserve, was used both for patient stratification and longitudinal assessment because postoperative AMH decline is strongly influenced by baseline ovarian reserve (10).

## Methods

### Study design and participants

This single-center, parallel-group randomized controlled trial was conducted to evaluate whether vasopressin injection during laparoscopic cystectomy could reduce ovarian reserve loss in women with unilateral ovarian endometriomas. Participants were randomly assigned in a 1:1 ratio to either the vasopressin or control group. The trial was registered at ClinicalTrials.gov (NCT04372836). The first participant was enrolled on October 29, 2018.

Women scheduled to undergo laparoscopic surgery for ovarian endometrioma between September 2018 and July 2021 at Severance Hospital, Yonsei University College of Medicine (Seoul, Republic of Korea), were screened for eligibility. Inclusion criteria were: age 19–45 years; serum AMH level between 0.5 and 7.0 ng/mL (11); unilateral ovarian endometrioma; regular menstrual cycles during the preceding 6 months; no previous surgery for endometriosis; no hormonal treatment for endometriosis, including oral contraceptives, progestins, or gonadotropin-releasing hormone agonists, within 3 months before surgery; and no significant medical comorbidities.

The study protocol was approved by the Institutional Review Board of Yonsei University College of Medicine (approval number: 4-2018-0732; September 15, 2018). Written informed consent was obtained from all participants prior to study enrollment. The study was conducted in accordance with the Declaration of Helsinki.

All participants had unilateral unilocular or bilocular ovarian endometriomas diagnosed by transvaginal ultrasonography and/or magnetic resonance imaging (MRI) (12). Endometrioma size was defined as the mean of the maximum cyst diameter and its perpendicular diameter measured on imaging studies (13). For bilocular endometriomas, cyst size was calculated as the sum of the mean diameters of the individual cyst locules.

### Randomization

Participants were stratified according to baseline serum AMH levels into a low-AMH group (0.5–<3.0 ng/mL) and a high-AMH group (3.0–<7.0 ng/mL). Within each stratum, participants were randomly assigned in a 1:1 ratio to the vasopressin or control group using a computer-generated block randomization scheme with a block size of four. A unique randomization identification number was assigned to each participant at enrollment according to the screening sequence. The allocation sequence was generated by an independent statistician using a computer-generated block randomization scheme with a block size of four and stratification by baseline AMH level.

### Procedures

Laparoscopic cystectomy was performed primarily through a single 12-mm subumbilical port. An additional 5-mm lower abdominal port was placed when required in cases with severe pelvic adhesions. After a sharp incision was made on the cyst surface, ovarian cyst enucleation was performed using a traction–countertraction technique with two atraumatic grasping forceps. Excised cyst specimens were submitted for histopathological confirmation.

Hemostasis was achieved using bipolar coagulation and, when necessary, suturing to minimize thermal injury to the ovarian cortex. Although suturing was the preferred method of hemostasis, bipolar coagulation was applied when persistent bleeding could not be adequately controlled. All procedures were performed by two experienced surgeons (S.W.K. and J.H.L.).

In the vasopressin group, a diluted vasopressin solution (20 IU vasopressin diluted in 50 mL normal saline; Hanlim Pharm, Republic of Korea) was injected into the plane between the cyst wall and ovarian cortex before cyst enucleation. Using an 18-gauge aspiration needle (33.5 × 3.5 mm; Wisap Medical, Germany), 10–30 mL of solution was administered until adequate hydrodissection and separation of the cyst wall from the surrounding ovarian tissue were achieved. In the control group, cystectomy was performed without vasopressin injection.

Additional endometriotic lesions identified within the pelvis, including superficial peritoneal and deep infiltrating lesions, were excised whenever feasible using shaving, sharp dissection, or electrofulguration.

To reduce the risk of postoperative recurrence, all participants received hormonal suppression therapy for at least 12 months after surgery. Treatment consisted of a gonadotropin-releasing hormone (GnRH) agonist (leuprorelin acetate; Takeda, Republic of Korea) administered for 3–6 months, followed by dienogest 2 mg daily (Bayer AG, Germany).

### Outcomes

The primary outcome was the change in serum AMH level from baseline to 12 months after surgery. Serum AMH levels were measured preoperatively and at 3, 6, and 12 months postoperatively to evaluate longitudinal changes in ovarian reserve.

For AMH analysis, peripheral blood samples were collected in serum-separating tubes and centrifuged at 1,000 × g for 10 min within 2 h of collection. Serum AMH concentrations were measured using an automated electrochemiluminescence assay (Cobas® 8,000 analyzer; Roche Diagnostics International Ltd., Rotkreuz, Switzerland).

Secondary outcomes included operative parameters, specifically the time required for cyst stripping and the time required to achieve hemostasis using bipolar coagulation. The severity of endometriosis was classified according to the revised American Society for Reproductive Medicine (rASRM) classification system (14).

Endometrioma recurrence was assessed by transvaginal ultrasonography 12 months after surgery. Recurrence was defined as the presence of a homogeneous hypoechoic cyst measuring >1 cm in diameter without detectable blood flow at the surgical site (15).

### Pathological analysis

Following cystectomy, excised endometrioma specimens were serially sectioned and submitted for histopathological evaluation. For cysts larger than 3 cm in greatest diameter, representative tissue sections were selected for analysis.

All hematoxylin and eosin (H&E)-stained slides containing cyst wall and adjacent ovarian tissue were independently reviewed by a gynecologic pathologist (E.P.) who was blinded to the participants' clinical characteristics and treatment allocation.

The mean thickness of the cyst wall, the mean thickness of ovarian tissue attached to the cyst wall (when present), and *the proportion of ovarian tissue within the excised specimen* area were measured and recorded.

### Statistical analysis

The sample size was calculated to detect a difference in the decline of serum AMH levels between the vasopressin and control groups. Because no previous study had evaluated the effect of intraoperative vasopressin injection on postoperative AMH decline following endometrioma cystectomy at the time of study design, sample size estimation was based on a pilot dataset obtained from nine women. The pilot data demonstrated smaller postoperative reductions in AMH levels among women who received vasopressin injection.

To ensure adequate statistical power, conservative effect size assumptions were adopted. In the low-AMH stratum, the expected difference (*δ*) and standard deviation (*σ*) were both set at 0.5 ng/mL, whereas in the high-AMH stratum, *δ* and *σ* were set at 1.0 ng/mL. Based on a two-sided *α* level of 0.05% and 80% power, 68 evaluable participants (17 per treatment subgroup within each AMH stratum) were required. Assuming a 10% dropout rate, a total sample size of 75 participants was planned.

Baseline demographic, clinical, and surgical characteristics were summarized using descriptive statistics. Continuous variables were compared using independent-samples t-tests, whereas categorical variables were analyzed using the chi-square test or Fisher's exact test, as appropriate.

The primary outcome, the change in serum AMH level at 12 months after surgery, was compared between groups using independent-samples t-tests and analysis of covariance (ANCOVA). ANCOVA was performed to improve the precision of the estimated treatment effect by adjusting for baseline AMH and cyst size, which were prespecified clinically relevant covariates. Longitudinal changes in serum AMH over the four follow-up time points were analyzed using linear mixed-effects models to evaluate group-by-time interactions. *Post hoc* analyses were performed for multiple comparisons when appropriate.

The primary analysis followed the intention-to-treat (ITT) principle without imputation of missing values. Sensitivity analyses using the last observation carried forward (LOCF) approach and per-protocol (PP) analyses were performed and are presented in the Supplementary Materials. All statistical analyses were conducted using SAS version 9.4 (SAS Institute Inc., Cary, NC, USA). A two-sided *p* value <0.05 was considered statistically significant.

## Results

A total of 75 women were enrolled and randomized, with 37 assigned to the vasopressin group and 38 to the control group. All participants underwent baseline assessment and postoperative follow-up for up to 12 months, with the final study visit completed in July 2021. Eleven participants were lost to follow-up or excluded because of protocol deviations, resulting in 60 women completing the study. Participant flow through the trial is summarized in Figure 1.

**Figure 1:**
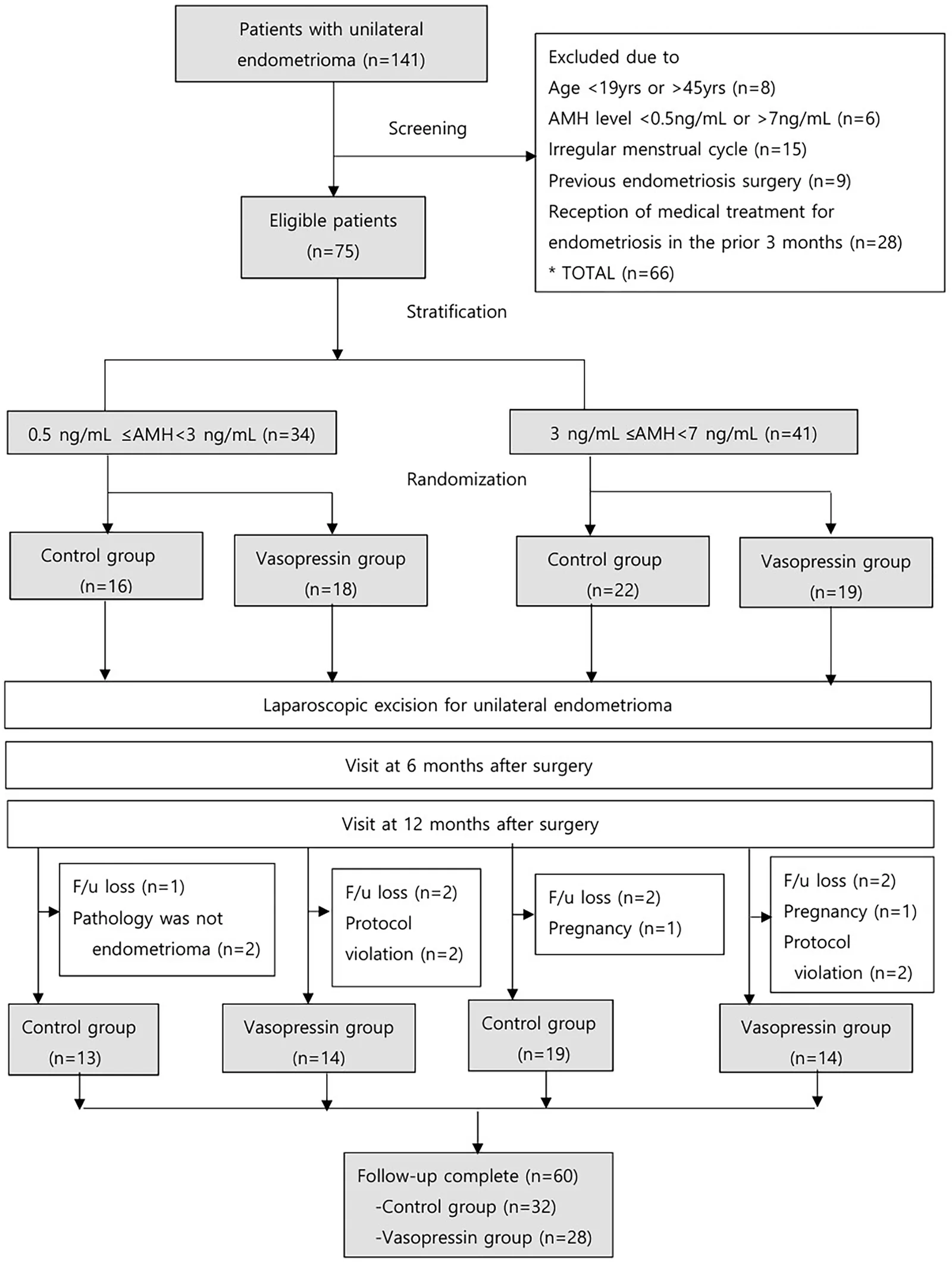
Flow diagram of the randomized controlled trial. A total of 141 patients with unilateral ovarian endometrioma were screened for eligibility. After exclusions based on age, AMH levels, menstrual irregularity, history of prior endometriosis surgery, and recent hormonal treatment, 75 eligible patients were enrolled. These participants were stratified by baseline serum AMH level (0.5–3 ng/mL and 3–7 ng/mL) and then randomized to either the vasopressin injection group or the control group. All patients underwent laparoscopic cystectomy and were scheduled for postoperative follow-up at 6 and 12 months. Losses to follow-up, pregnancies, protocol violations, and non-endometrioma pathology findings were documented. A total of 60 patients completed the study per protocol and were included in the per-protocol analysis, while all randomized patients (*n* = 75) were included in the primary intention-to-treat (ITT) analysis.

Baseline demographic and clinical characteristics were comparable between the two groups (Table 1). No significant differences were observed in age, body weight, baseline serum AMH level, endometrioma size, cyst characteristics, or rASRM stage. Intraoperatively, vasopressin injection significantly reduced both cyst stripping time (8.9 ± 7.9 vs. 14.4 ± 13.7 min, *p* = 0.054) and hemostasis time (2.2 ± 2.5 vs. 6.5 ± 6.8 min, *p* = 0.002) compared with the control group. Postoperative hemoglobin decline tended to be smaller in the vasopressin group, although the difference did not reach statistical significance. No recurrence of endometrioma was observed in either group during the 12-month follow-up period.

**Table 1:** Baseline characteristics of the study population, intraoperative findings, surgical procedures, and postoperative findings.

Variables	Control group (*n* = 38)	Vasopressin group (*n* = 37)	Overall (*n* = 75)	*p*-value
Age, years	32.4 ± 5.8	33.4 ± 5.4	32.9 ± 5.6	0.425
Height, cm	161.5 ± 4.7	162.3 ± 5.3	161.9 ± 5.0	0.488
Weight, kg	55.1 ± 6.6	56.1 ± 7.6	55.6 ± 7.1	0.524
Type of unilateral endometrioma				0.736
Bilocular	5 (13.16)	4 (10.81)	9 (12.00)	
Unilocular	33 (86.84)	33 (89.19)	66 (88.00)	
Size of unilateral endometrioma, cm	5.0 ± 1.7	5.1 ± 2.2	5.0 ± 1.9	0.765
Baseline AMH, ng/mL	3.7 ± 2.0	3.3 ± 1.8	3.5 ± 1.9	0.370
AMH group, *n* (%)				0.569
0.5 ng/mL ≤ AMH<3 ng/mL	16 (42.11)	18 (48.65)	34 (45.33)	
3 ng/mL ≤ AMH <7 ng/mL	22 (57.89)	19 (51.35)	41 (54.67)	
Dysmenorrhea, VAS score	5.0 ± 2.7	5.0 ± 2.6	5.0 ± 2.6	>0.999
Endometrial phase at the time of surgery				0.112
proliferative	22 (57.89)	26 (70.27)	48 (64.00)	
secretory	16 (42.11)	10 (29.73)	26 (36.00)	
Time required for cyst stripping, min	14.4 ± 13.7	8.9 ± 7.9	11.5 ± 11.3	0.054
Time required for coagulate bleeding, min	6.5 ± 6.8	2.2 ± 2.5	4.2 ± 5.4	0.002
Endometriosis stages according to the revised ASRM classification, *n* (%)				
I, II	0	0	0	0.946
III	22 (57.89)	24 (64.86)	46 (61.33)	
IV	16 (42.11)	13 (35.14)	29 (38.67)	
Revised ASRM classification total score^a^	28 (20–112)	27 (21–102)	28 (20–112)	0.543
Hb decrease after surgery, g/dL	1.9 ± 0.9	1.5 ± 0.7	1.7 ± 0.8	0.073

AMH, anti-Müllerian hormone; ASRM, American Society for Reproductive Medicine; Hb, hemoglobin; VAS, visual analogue scale.

a The Mann–Whitney U test was used (mean ± standard deviation).

The primary outcome analysis demonstrated no significant difference in postoperative ovarian reserve decline between the two groups. The mean change in serum AMH level at 12 months after surgery was −0.96 ± 1.11 ng/mL in the vasopressin group and −1.14 ± 0.92 ng/mL in the control group (*p* = 0.488) (Table 2). Subgroup analyses stratified by baseline AMH level (0.5–<3.0 ng/mL and 3.0–<7.0 ng/mL) likewise showed no significant differences between groups. These findings remained unchanged after adjustment for baseline AMH level and endometrioma size using ANCOVA. A sensitivity analysis excluding the two participants without histopathological confirmation of endometrioma yielded similar results and did not alter the overall conclusions (Supplementary Table S6).

**Table 2:** Changes in AMH value 12 months after excision of unilateral endometriomas.

Model	Subgroup	Control group (*n* = 38)	Vasopressin group (*n* = 37)	Mean Difference (95% CI*)*	*p*-value
Crude^a^	Overall, ng/mL	−1.14 ± 0.92	−0.96 ± 1.11	−0.19(−0.72–0.35)	0.488
	0.5≤ AMH < 3, ng/mL	−0.67 ± 0.62	−0.47 ± 0.83	−0.20(−0.78–0.39)	0.491
	3≤ AMH < 7, ng/mL	−1.48 ± 0.97	−1.44 ± 1.18	−0.04(−0.81–0.73)	0.915
Model 1^b^	Overall, ng/mL	−1.11 (0.17)	−0.99 (0.18)	−0.12(−0.63–0.39)	0.631
	0.5≤ AMH < 3, ng/mL	−0.67 (0.18)	−0.47 (0.18)	−0.20(−0.74–0.33)	0.437
	3≤ AMH < 7, ng/mL	−1.48 (0.25)	−1.44 (0.28)	−0.04(−0.82–0.74)	0.909

AMH, anti-Müllerian hormone; ANCOVA, analysis of covariance; SD, standard deviation; SE, standard error.

a Independent 2-sample t-test, mean ± SD.

b Model 1: adjusted for baseline AMH value and endometrioma size: ANCOVA/mean (SE).

Longitudinal analysis using linear mixed-effects models demonstrated a significant decline in serum AMH levels after surgery in both the vasopressin and control groups, with partial recovery observed during follow-up (Table 3). However, no significant group-by-time interaction was identified in the overall cohort (p for group×time = 0.816), indicating that the pattern of AMH change over time did not differ between the two groups. Similarly, no significant differences in AMH levels were observed between groups at any individual time point.

**Table 3:** AMH values over time after excision of unilateral endometriomas.

Subgroup	Group	Time after surgery				*p*_group x time_^a^
		Baseline	3 months	6 months	12 months	
Overall	Control group, ng/mL	3.58 (0.32)	2.12 (0.24)	2.41 (0.28)	2.46 (0.30)	0.816
	Vasopressin group, ng/mL	3.27 (0.32)	1.76 (0.24)	1.93 (0.28)	2.20 (0.30)	
	*p*-value^b^	0.490	0.306	0.225	0.553	
0.5≤ AMH < 3 ng/mL (*n* = 34)	Control group, ng/mL	1.54 (0.18)	0.83 (0.16)	0.85 (0.18)	0.90 (0.23)	0.923
	Vasopressin group, ng/mL	1.73 (0.16)	1.03 (0.16)	1.11 (0.17)	1.24 (0.22)	
	*p*-value	0.447	0.381	0.308	0.286	
3≤ AMH < 7 ng/mL (*n* = 41)	Control group, ng/mL	4.97 (0.29)	2.94 (0.32)	3.55 (0.37)	3.48 (0.38)	0.556
	Vasopressin group, ng/mL	4.72 (0.31)	2.48 (0.35)	2.67 (0.39)	3.13 (0.42)	
	*p*-value	0.560	0.331	0.109	0.532	

AMH, anti-Müllerian hormone; SE, standard error.

The estimates are expressed as the mean (SE).

a *p*_group×time_: *p*-value for difference in AMH value between vasopressin group and control group at 3 time points, calculated using the linear mixed model.

b Comparison between vasopressin group and control group at each time point.

Subgroup analyses stratified by baseline AMH level yielded similar findings. No significant group-by-time interaction was observed in either the low-AMH subgroup (*p* = 0.923) or the high-AMH subgroup (*p* = 0.556). *Post hoc* analyses demonstrated that AMH levels were significantly lower at 3, 6, and 12 months than at baseline in both the overall cohort and the AMH-stratified subgroups (Supplementary Table S1). Although a modest recovery in AMH levels was observed after the initial postoperative decline, AMH concentrations remained significantly below baseline values throughout the follow-up period.

Sensitivity analyses using the LOCF method yielded findings consistent with those of the primary intention-to-treat analysis (Supplementary Tables S2, 3). Per-protocol analyses produced similar results, showing no significant differences in postoperative AMH decline between the vasopressin and control groups in either the overall cohort or the AMH-stratified subgroups (Supplementary Tables S4, 5). Likewise, no significant group-by-time interaction was observed in longitudinal analyses of the PP population (*p* = 0.906).

Histopathological evaluation revealed no significant differences between the vasopressin and control groups in mean cyst wall thickness, mean thickness of ovarian tissue attached to the cyst wall, or the proportion of ovarian tissue within the excised specimen (all *p* > 0.05; Table 4 and Supplementary Figure S1).

**Table 4:** The thickness and proportions of endometriotic cyst wall and attached ovarian tissue.

Histopathologic findings	Control group (*n* = 38)	Vasopressin group (*n* = 37)	*p*-value
Endometrioma cyst wall thickness (mean, mm)	1.6 ± 0.8	1.6 ± 0.7	0.958
Ovarian tissue thickness (mean, mm)	0.9 ± 0.8	0.7 ± 0.6	0.205
Proportion of ovarian tissue (%)	34.9 ±29.4	33.8 ± 29.7	0.876

Values are presented as mean ± standard deviation.

## Discussion

In this randomized controlled trial, vasopressin injection improved surgical efficiency by reducing cyst stripping and hemostasis times; however, it did not preserve ovarian reserve following laparoscopic cystectomy for unilateral ovarian endometrioma. Although AMH levels showed partial recovery after the initial postoperative decline, they remained significantly lower than baseline values at 12 months in both groups. Furthermore, no significant differences in AMH levels were observed between the vasopressin and control groups at any follow-up time point, regardless of baseline ovarian reserve. These findings suggest that reducing intraoperative bleeding and electrocoagulation exposure alone may be insufficient to prevent postoperative ovarian reserve loss associated with endometrioma surgery.

Previous studies have demonstrated that ovarian endometriomas themselves may adversely affect ovarian reserve and function (16). Mechanical compression of the surrounding ovarian cortex can disrupt tissue architecture, impair vascularization, and compromise local blood supply (1). In addition, endometriotic cyst fluid contains high concentrations of reactive oxygen species, inflammatory mediators, proteolytic enzymes, and free iron, all of which may contribute to chronic ovarian injury (17, 18). These mechanisms may explain why women with endometriomas often exhibit lower AMH levels than age-matched women without the disease (5, 19, 20).

Laparoscopic cystectomy remains the standard surgical treatment for ovarian endometriomas because it is more effective than drainage and coagulation in reducing recurrence and alleviating endometriosis-associated pain (12, 21). Although high-quality randomized evidence remains limited, excisional surgery has also been associated with improved spontaneous pregnancy rates in subfertile women with endometriomas larger than 3–4 cm (12, 22, 23). However, these benefits must be balanced against the potential risk of ovarian injury. Muzii et al. demonstrated that endometriotic cysts are more likely than other benign ovarian cysts to contain adjacent healthy ovarian tissue, increasing the risk of inadvertent tissue removal during cystectomy (24). In addition, thermal damage caused by bipolar coagulation and impairment of ovarian vascularization following excision may contribute to the postoperative decline in ovarian reserve, as reflected by reductions in serum AMH levels (1, 25).

Several strategies have therefore been proposed to minimize ovarian damage during cystectomy. Among these, suturing and hemostatic sealants have been used as alternatives to bipolar coagulation in an effort to reduce thermal injury to the ovarian cortex and its vascular network (26). Vasopressin injection represents another potential ovarian-sparing strategy by facilitating hydrodissection between the cyst wall and ovarian stroma and by reducing intraoperative bleeding (6–9).

Evidence regarding the effectiveness of vasopressin-assisted cystectomy remains inconclusive. Among the randomized trials published in English, Qiong-Zhen et al. reported reduced use of bipolar coagulation and less postoperative elevation of follicle-stimulating hormone levels following vasopressin injection, suggesting improved preservation of ovarian function (6). In contrast, Ghafarnejad et al. observed no significant difference in postoperative antral follicle count between the vasopressin and control groups (8). More recently, Alborzi et al. reported that although vasopressin reduced intraoperative bleeding, it did not significantly attenuate postoperative AMH decline, findings that closely mirror those of the present study (9).

A recent meta-analysis of seven randomized controlled trials concluded that vasopressin-assisted cystectomy may have a beneficial effect on ovarian reserve preservation (27). However, substantial clinical heterogeneity existed among the included studies, particularly with respect to endometrioma laterality, baseline ovarian reserve, outcome measures, and duration of follow-up. In contrast, the present trial was restricted to women with unilateral endometriomas and incorporated stratification according to baseline AMH levels, with longitudinal assessment of ovarian reserve for up to 12 months after surgery. Despite demonstrating significant reductions in cyst stripping and hemostasis times, our findings suggest that these procedural advantages do not translate into measurable preservation of ovarian reserve.

Consistent with the findings of Alborzi et al. (9), vasopressin injection did not attenuate the postoperative decline in AMH levels following endometrioma cystectomy. This observation was also consistent with the histopathological findings of the present study. The mean thickness of the cyst wall, the mean thickness of ovarian tissue attached to the cyst wall, and the proportion of ovarian tissue within the excised specimen did not differ significantly between the vasopressin and control groups. Together, these findings suggest that although vasopressin may facilitate surgical dissection and reduce intraoperative bleeding through its hydrodissection effect, it may not substantially influence the amount of ovarian tissue removed during cystectomy or postoperative ovarian reserve when the procedure is performed by experienced surgeons.

Several factors should also be considered when interpreting the absence of a significant difference in postoperative AMH decline between the two groups. First, all participants received postoperative hormonal suppression therapy to reduce the risk of recurrence. Both gonadotropin-releasing hormone (GnRH) agonists and dienogest have been reported to suppress ovarian activity and may influence serum AMH concentrations (28, 29). In particular, a previous Korean study reported amenorrhea in approximately 90% of women receiving dienogest, suggesting substantial ovarian suppression (30). Because the same postoperative hormonal suppression regimen was applied to both groups, this factor is unlikely to have biased the primary between-group comparison. However, it may have altered the absolute trajectory of AMH recovery and reduced the magnitude of detectable differences between groups, particularly during the early postoperative period when GnRH agonist therapy was administered. The use of the 12-month postoperative AMH measurement as the primary endpoint also helped minimize the influence of transient hormonal suppression compared with earlier postoperative assessments. Nevertheless, postoperative hormonal treatment should be considered when interpreting the longitudinal AMH changes observed during follow-up.

Second, although the target sample size was calculated to accommodate an anticipated dropout rate of approximately 10%, participant attrition exceeded expectations, reducing the number of participants available for the per-protocol analysis. This reduction in sample size may have limited the statistical power to detect small but potentially clinically meaningful between-group differences. Accordingly, the possibility of a type II error cannot be excluded, and the absence of a statistically significant difference should not be interpreted as evidence of equivalence between the two interventions. Future adequately powered multicenter studies are warranted to confirm these findings and determine whether modest treatment effects exist. Nevertheless, the consistency of findings across the intention-to-treat, per-protocol, and sensitivity analyses supports the overall robustness of our findings.

Finally, two participants included in the intention-to-treat analysis were subsequently found to have dermoid cysts rather than endometriomas on final histopathological examination (Figure 1). Because dermoid cystectomy is generally associated with less postoperative decline in AMH than endometrioma cystectomy (31), inclusion of these participants may have introduced a small degree of outcome misclassification. However, given the limited number of affected cases and the concordant findings observed in the per-protocol analysis, the overall impact on the study conclusions is likely to have been minimal.

### Limitation

The present study has several strengths. Participants were stratified according to baseline AMH level, one of the strongest determinants of postoperative ovarian reserve decline, and only women with unilateral endometriomas were included, thereby reducing clinical heterogeneity and allowing adjustment for endometrioma size. In addition, histopathological evaluation of the excised specimens provided mechanistic support for the clinical findings by demonstrating no significant differences in the amount of ovarian tissue removed between groups.

Several limitations should also be acknowledged. First, the follow-up period was limited to 12 months, precluding assessment of long-term reproductive outcomes such as spontaneous conception, assisted reproductive technology outcomes, and live birth. Second, postoperative hemostasis was achieved using a combination of suturing and bipolar coagulation rather than suturing alone. Because thermal injury from electrocoagulation may adversely affect ovarian reserve, its use may have attenuated any potential ovarian-sparing effect of vasopressin. However, this approach reflects routine clinical practice and is consistent with the methodology used in previous randomized controlled trials evaluating vasopressin-assisted cystectomy (6, 8, 9). Moreover, bipolar coagulation was selectively applied only when adequate hemostasis could not be achieved with suturing alone.

## Recommendation and conclusion

Vasopressin-assisted laparoscopic cystectomy improved surgical efficiency by reducing cyst stripping and hemostasis times but did not attenuate postoperative ovarian reserve decline. These findings suggest that reducing intraoperative bleeding and electrocoagulation exposure alone may be insufficient to preserve ovarian reserve after endometrioma surgery.

## Data Availability

The original contributions presented in the study are included in the article/Supplementary Material, further inquiries can be directed to the corresponding author.

## Supplementary material

Supplementary Figure 1 Representative images of full thickness of the cyst wall of endometriomas (A) with no attached normal ovarian tissue and (B) with presence of attached ovarian tissue (hematoxylin and eosin; magnification ×10).
