# Supplementary material for: Impact of vasopressin-assisted laparoscopic endometrioma excision on ovarian reserve: a randomized controlled trial
Source: Front Reprod Health. 2026 Oct 2;8:1906041. doi: 10.3389/frph.2026.1906041 (PMC13671997; doi:10.3389/frph.2026.1906041)
Supplement: Supplementary file 1 [file Table1.docx]

**Supplementary Table 1. Post hoc analyses (linear mixed model) of changes in AMH values between time points**

| Subgroup | Group | Time after surgery | | | | |
| --- | --- | --- | --- | --- | --- | --- |
|  |  | 3 months  -initial | 6 months  - initial | 12 months  - initial | 6 months  - 3 months | 12 months  - 6 months |
| Overall | Control group, ng/mL | -1.46(0.20) ^a^ | -1.17(0.21) ^a^ | -1.13(0.19) ^a^ | 0.30(0.15) ^d^ | 0.04(0.17) ^d^ |
|  | Vasopressin group, ng/mL | -1.50(0.20) ^a^ | -1.34(0.22) ^a^ | -1.07(0.19) ^a^ | 0.16(0.16) ^d^ | 0.28(0.18) ^d^ |
|  | *p* _group x time_ | .893 | .567 | .824 | .535 | .345 |
| 0.5 ≤ AMH < 3, ng/mL  (n=34) | Control group, ng/mL | -0.72(0.15) ^a^ | -0.69(0.17) ^a^ | -0.65(0.19) ^b^ | 0.03(0.15) ^d^ | 0.04(0.11) ^d^ |
|  | Vasopressin group, ng/mL | -0.70(0.14) ^a^ | -0.62(0.16) ^a^ | -0.49(0.18) ^b^ | 0.08(0.15) ^d^ | 0.18(0.10) ^d^ |
|  | *p* _group x time_ | .946 | .753 | .555 | .786 | .569 |
| 3 ≤ AMH < 7, ng/mL  (n=41) | Control group, ng/mL | -2.03(0.27) ^a^ | -1.42(0.32) ^a^ | -1.49(0.25) ^a^ | 0.61(0.24) ^c^ | -0.07(0.31)^d^ |
|  | Vasopressin group, ng/mL | -2.24(0.29) ^a^ | -2.05(0.34) ^a^ | -1.59(0.28) ^a^ | 0.19(0.26) ^d^ | 0.46(0.34) ^d^ |
|  | *p* _group x time_ | .590 | .183 | .782 | .247 | .258 |

Abbreviations: AMH, anti-Müllerian hormone; SE, standard error.

Estimates are expressed as the mean (SE).

^a^ *p* < 0.001, ^b^ *p* < 0.01, ^c^ *p* < 0.05, ^d^ *p* > 0.05: *p*-value for difference of AMH values between 2 time points within the group, calculated by linear mixed model.

**Supplementary Table 2. Changes in AMH value 12 months after surgery (ITT analysis, LOCF)**

| **Model** | **Subgroup** | **Control group (n=38)** | **Vasopressin group (n=37)** | ***p*-value** |
| --- | --- | --- | --- | --- |
| Crude^a^ | Overall, ng/mL | -1.01±0.98 | -1.06±1.32 | .853 |
|  | 0.5≤ AMH < 3, ng/mL | -0.61±0.60 | -0.41±0.74 | .408 |
|  | 3≤ AMH < 7, ng/mL | -1.28±1.10 | -1.67±1.46 | .335 |
| Model 1^b^ | Overall, ng/mL | -0.96(0.17) | -1.10(0.17) | .580 |
|  | 0.5≤ AMH < 3, ng/mL | -0.63(0.16) | -0.40(0.15) | .299 |
|  | 3≤ AMH < 7, ng/mL | -1.23(0.27) | -1.73(0.29) | .226 |

Abbreviations: AMH, Anti-Müllerian Hormone; ANCOVA, analysis of covariance; SD, standard deviation; SE, standard error; ITT, intent-to-treat; LOCF, last observation carried forward.

^a^ Independent two-sample t-test, mean ± SD

^b^ Model 1: adjusted for baseline AMH value and endometrioma size: ANCOVA/mean (SE)

**Supplementary Table 3. AMH values over time after excision of unilateral endometriomas (ITT analysis, LOCF)**

| Subgroup | Group | Time after surgery | | | | *p* _group x time_^a^ |
| --- | --- | --- | --- | --- | --- | --- |
|  |  | Baseline | 3 months | 6 months | 12 months |  |
| Overall | Control group, ng/mL | 3.58(0.32) | 2.66(0.30) | 2.50(0.28) | 2.57(0.29) | .918 |
|  | Vasopressin group, ng/mL | 3.27(0.32) | 2.38(0.30) | 2.03(0.28) | 2.21(0.29) |  |
|  | *p*-value^b^ | .490 | .504 | .237 | .380 |  |
| 0.5≤ AMH < 3, ng/mL  (n=34) | Control group, ng/mL | 1.54(0.18) | 0.91(0.19) | 0.87(0.21) | 0.93(0.23) | .665 |
|  | Vasopressin group, ng/mL | 1.73(0.16) | 1.31(0.17) | 1.22(0.19) | 1.32(0.21) |  |
|  | *p*-value^b^ | .447 | .121 | .222 | .23 |  |
| 3≤ AMH < 7, ng/mL  (n=41) | Control group, ng/mL | 4.97(0.29) | 3.86(0.34) | 3.61(0.33) | 3.70(0.35) | .644 |
|  | Vasopressin group, ng/mL | 4.72(0.31) | 3.39(0.37) | 2.80(0.36) | 3.05(0.38) |  |
|  | *p*-value^b^ | .560 | .358 | .107 | .222 |  |

Abbreviations: AMH, Anti-Müllerian Hormone; SE, standard error; ITT, intent-to-treat; LOCF, last observation carried forward.

Estimates are expressed as the mean (SE).

^a^ *p* _group x time_: *p*-value for difference in AMH values between vasopressin group and control group at 3 time points, calculated using the linear mixed model.

^b^ Comparison between vasopressin group and control group at each time point

**Supplementary Table 4. Changes in AMH value 12 months after excision of unilateral endometriomas (per-protocol analysis)**

| **Model** | **Subgroup** | **Control group (n=32)** | **Vasopressin group (n=32)** | ***p*-value** |
| --- | --- | --- | --- | --- |
| Crude^a^ | Overall, ng/mL | -1.08±1.05 | -1.03±1.12 | .906 |
|  | 0.5≤ AMH < 3, ng/mL | -0.79±0.66 | -0.44±0.90 | .321 |
|  | 3≤ AMH < 7, ng/mL | -1.39±1.33 | -1.85±0.89 | .420 |
| Model 1^b^ | Overall, ng/mL | -1.02(0.24) | -1.09(0.24) | .821 |
|  | 0.5≤ AMH < 3, ng/mL | -0.74(0.24) | -0.49(0.23) | .451 |
|  | 3≤ AMH < 7, ng/mL | -1.27(0.39) | -1.98(0.41) | .252 |

Abbreviations: AMH, Anti-Müllerian Hormone; ANCOVA, analysis of covariance; SD, standard deviation; SE, standard error.

^a^ Independent two-sample t-test, mean ± SD

^b^ Model 1: adjusted for baseline AMH value and endometrioma size: ANCOVA/mean (SE)

**Supplementary Table 5. AMH values over time after excision of unilateral endometriomas (per-protocol analysis)**

| Subgroup | Group |  | | Time after surgery | | | | *p* _group x time_^a^ |
| --- | --- | --- | --- | --- | --- | --- | --- | --- |
|  |  | Baseline | 3 months | | 6 months | | 12 months |  |
| Overall | Control group, ng/mL | 3.55(0.47 | 2.11(0.36) | | 2.49(0.44) | 2.48(0.44) | | .907 |
|  | Vasopressin group, ng/mL | 2.97(0.47) | 1.68(0.36) | | 1.89(0.44) | 1.93(0.44 | |  |
|  | *p*-value^b^ | .381 | .397 | | .337 | .385 | |  |
| 0.5≤AMH <3 ng/mL (n=29) | Control group, ng/mL | 1.76(0.19) | 0.89(0.17) | | 0.89(0.23) | 0.96(0.30) | | .682 |
|  | Vasopressin group, ng/mL | 1.70(0.18) | 1.04(0.17) | | 1.17(0.22) | 1.26(0.29) | |  |
|  | *p*-value^b^ | .813 | .550 | | .405 | .493 | |  |
| 3≤ AMH < 7 ng/mL (n=35) | Control group, ng/mL | 5.55(0.50) | 3.46(0.54) | | 4.27(0.64) | 4.16(0.63) | | .529 |
|  | Vasopressin group, ng/mL | 4.71(0.53) | 2.56(0.57) | | 2.88(0.68) | 2.86(0.67) | |  |
|  | *p*-value^b^ | .267 | .266 | | .154 | .181 | |  |

Abbreviations: AMH, anti-Müllerian hormone; SE, standard error.

Estimates are expressed as the mean (SE).

^a^ *p* _group x time_: *p*-value for difference in AMH value between vasopressin group and control group at 3 time points, calculated using the linear mixed model.

^b^ Comparison between vasopressin group and control group at each time point.

**Supplementary Table 6. Sensitivity analysis of changes in AMH value 12 months after exclusion of participants without histopathological confirmation of endometrioma.**

| **Model** | **Subgroup** | **Control group (n=38)** | **Vasopressin group (n=37)** | **Mean Difference (95% CI*)*** | ***p*-value** |
| --- | --- | --- | --- | --- | --- |
| Crude^a^ | Overall, ng/mL | -1.18±0.92 | -0.96±1.11 | -0.22 (-0.76-0.32) | .413 |
|  | 0.5≤ AMH < 3, ng/mL | -0.72±0.63 | -0.47±0.83 | -0.25 (-0.85-0.36) | .406 |
|  | 3≤ AMH < 7, ng/mL | -1.48±0.97 | -1.44±1.18 | -0.04 (-0.81-0.73) | .915 |
| Model 1^b^ | Overall, ng/mL | -1.14(0.18) | -1.00(0.19) | -0.14 (-0.66-0.38) | .585 |
|  | 0.5≤ AMH < 3, ng/mL | -0.68 (0.20) | -0.50(0.18) | -0.18 (-0.74-0.37) | .500 |
|  | 3≤ AMH < 7, ng/mL | -1.48 (0.25) | -1.44(0.28) | -0.04 (-0.82-0.74) | .909 |

Abbreviations: AMH, anti-Müllerian hormone; ANCOVA, analysis of covariance; SD, standard deviation; SE, standard error.

^a^ Independent 2-sample t-test, mean ± SD

^b^ Model 1: adjusted for baseline AMH value and endometrioma size: ANCOVA/mean (SE)
